# Supplementary material for: Deletion of wfs1 Impairs Oligodendrocyte Precursor Cells Dorsal Distribution and Myelination Through the wfs1-hmgcs1 Axis in Zebrafish
Source: Biology (Basel). 2026 Aug 16;15(16):1402. doi: 10.3390/biology15161402 (PMC13510151; doi:10.3390/biology15161402)
Supplement: Supplementary file 1 [file biology-15-01402-s001.zip › File S1-Transcriptome sequencing data and differentially expressed genes (DEGs) in wfs1-- zebrafish at 5 dpf/2.KEGG/wfs1KOvsWT/UP/src/dre00190.html]

dre00190


- dre:573619

- Up regulated genes

573619(9.34031008711337)

- dre:447914

- Up regulated genes

447914\_1(7.364607060603)

- dre:447942

- Up regulated genes

447942(1.89918802415248)

- dre:359840

- Up regulated genes

359840(3.81608997342262)
- dre:767750

- Up regulated genes

767750\_2(7.76955022165362) 767750(6.57768162814625)
- dre:359839

- Up regulated genes

359839(1.49784899389719)

- dre:447942

- Up regulated genes

447942(1.89918802415248)

- dre:767750

- Up regulated genes

767750\_2(7.76955022165362) 767750(6.57768162814625)

- dre:359840

- Up regulated genes

359840(3.81608997342262)
- dre:359839

- Up regulated genes

359839(1.49784899389719)

- dre:573619

- Up regulated genes

573619(9.34031008711337)

Close
